# Supplementary figures and images for: Amphibian diversity across three adjacent ecosystems in Área de Conservación Guanacaste, Costa Rica
Source: PeerJ. 2023 Nov 27;11:e16185. doi: 10.7717/peerj.16185 (PMC10688307; doi:10.7717/peerj.16185)

Abundance

Cacao

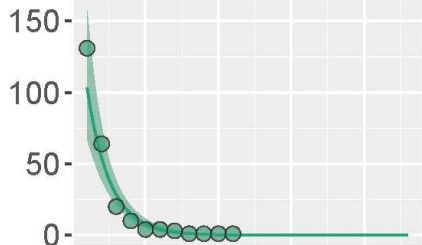

Maritza

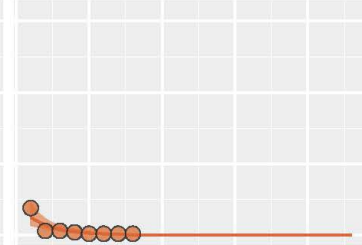

Murciélago

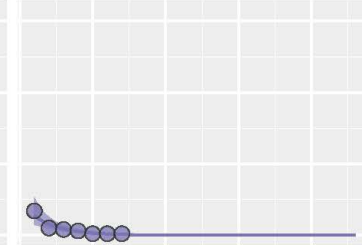

San Gerardo

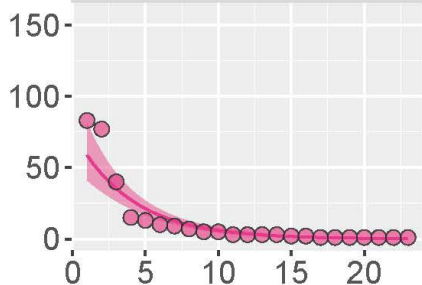

Santa Rosa

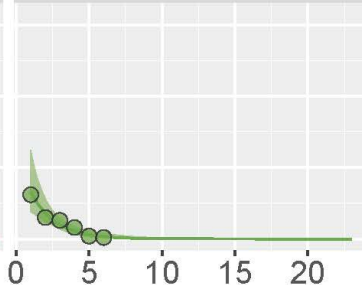

Rank

Supplement: Supplemental Information 1 [file peerj-11-16185-s001.pdf]
