# Supplementary material for: Amphibian diversity across three adjacent ecosystems in Área de Conservación Guanacaste, Costa Rica
Source: PeerJ. 2023 Nov 27;11:e16185. doi: 10.7717/peerj.16185 (PMC10688307; doi:10.7717/peerj.16185)
Supplement: Supplemental Information 2 — As well as the total number of individuals of each species caught at each sampling date. [file peerj-11-16185-s002.docx]

|  | | |
| --- | --- | --- |
| **Taxa** | **Cacao (1987/1988)** | **Cacao (2017)** |
| **Bufonidae** |  |  |
| *Rhinella horribilis* | 1 | - |
| **Centrolenidae** |  |  |
| *Hyalinobatrachium colymbiphyllum* | - | 4 |
| *Hyalinobatrachium fleischmanni* | 1 | - |
| *Espadarana prosoblepon* | 2 | - |
| **Craugastoridae** |  |  |
| *Craugastor bransfordii* | 2 | - |
| *Craugastor crassidigitus* | 3 | 64 |
| *Craugastor fitzingeri* | - | 4 |
| *Craugastor megacephalus* | 15 | 3 |
| *Craugastor andi* | 1 | - |
| *Craugastor melanosticus* | 4 | - |
| *Pristimantis ridens* | 3 | 10 |
| **Eleutherodactyldae** |  |  |
| *Diasporus diastema* | 2 | 1 |
| **Hylidae** |  |  |
| *Duellmanohyla uranochroa* | 1 | - |
| *Duellmanohyla rufioculis* | 27 | 131 |
| *Smilisca baudinii* | - | 1 |
| *Tlalocohyla loquax* | - | 1 |
| *Isthmohyla tica* | 1 | - |
| **Leptodactylidae** |  |  |
| *Engystomops pustulosus* | 1 | - |
| **Microhylidae** |  |  |
| *Hypopachus variolosus* | 2 | - |
| **Ranidae** |  |  |
| *Lithobates forreri* | 18 | 1 |
| *Lithobates warszewitschii* | 4 | 20 |
| **Plethodontidae** |  |  |
| *Nototriton guanacaste* | 18 | - |
| *Bolitoglossa robusta* | 1 | - |
| *Bolitoglossa subpalmata* | 1 | - |
| **Number of species** | 20 | 11 |
| **Number of individuals** | 108 | 240 |
