# Supplementary material for: Amphibian diversity across three adjacent ecosystems in Área de Conservación Guanacaste, Costa Rica
Source: PeerJ. 2023 Nov 27;11:e16185. doi: 10.7717/peerj.16185 (PMC10688307; doi:10.7717/peerj.16185)
Supplement: Supplemental Information 3 — Also includes the total number of individuals of each species caught at each site and in total at all sites. [file peerj-11-16185-s003.docx]

|  | | | | | | |  |
| --- | --- | --- | --- | --- | --- | --- | --- |
| **Taxa** | **Cacao** | **San Gerardo** | **Maritza** | **Murcielago** | **Santa Rosa** | **Total** | |
| **Bufonidae** |  |  |  |  |  |  | |
| *Incilius coccifer* | - | - | - | - | 1 | 1 | |
| *Incilius luetkenii* | - | - | - | 3 | - | 3 | |
| *Incilius melanochloris* | - | - | 1 | - | - | 1 | |
| *Rhaebo haematiticus* | - | 77 | - | - | - | 77 | |
| *Rhinella horribilis* | - | 3 | 1 | 17 | 31 | 52 | |
| **Centrolenidae** |  |  |  |  |  |  | |
| *Cochranella granulosa* | - | - | 2 | - | - | 2 | |
| *Hyalinobatrachium colymbiphyllum* | 4 | - | - | - | - | 4 | |
| *Teratohyla pulverata* | - | 15 | - | - | - | 15 | |
| *Teratohyla spinosa* | - | 5 | - |  | - | 5 | |
| **Craugastoridae** |  |  |  |  |  |  | |
| *Craugastor bransfordii* | - | 5 | - | - | - | 5 | |
| *Craugastor crassidigitus* | 64 | 13 | - | - | - | 77 | |
| *Craugastor fitzingeri* | 4 | 83 | 1 | 1 | - | 89 | |
| *Craugastor megacephalus* | 3 | 40 | - | - | - | 43 | |
| *Craugastor persimilis* | - | 3 | - | - | - | 3 | |
| *Craugastor ranoides* | - | - | - | 5 | - | 5 | |
| *Craugastor stejnegerianus* | - | - | 1 | - | - | 1 | |
| *Craugastor talamancae* | - | 1 | - | - | - | 1 | |
| *Pristimantis ridens* | 10 | 3 | - | - | - | 13 | |
| **Eleutherodactyldae** |  |  |  |  |  |  | |
| *Diasporus diastema* | 1 | - | - | - | - | 1 | |
| **Hylidae** |  |  |  |  |  |  | |
| *Dendropsophus ebraccatus* | - | 1 | - | - | - | 1 | |
| *Duellmanohyla rufioculis* | 131 | - | 3 | - | - | 134 | |
| *Isthmohyla pseudopuma* | - | 2 | - | - | - | 2 | |
| *Scinax boulengeri* | - | 1 | - | - | - | 1 | |
| *Scinax elaeochroa* | - | 1 | - | - | - | 1 | |
| *Scinax staufferi* | - | 1 | - | - | - | 1 | |
| *Smilisca baudinii* | 1 | 10 | - | 4 | - | 15 | |
| *Smilisca puma* | - | 1 | - | - | - | 1 | |
| *Smilisca sordida* | - | 7 | - | - | - | 7 | |
| *Tlalocohyla loquax* | 1 | - | - | - | - | 1 | |
| *Trachycephalus typhonius* | - | - | 3 | 1 | 2 | 6 | |
| **Leptodactylidae** |  |  |  |  |  |  | |
| *Engystomops pustulosus* | - | - | - | 1 | 15 | 16 | |
| *Leptodactylus savagei* | - | 2 | - | - | - | 2 | |
| **Microhylidae** |  |  |  |  |  |  | |
| *Hypopachus variolosus* | - | - | - | - | 8 | 8 | |
| **Phyllomedusidae** |  |  |  |  |  |  | |
| *Agalychnis callidryas* | - | 1 | - | - | - | 1 | |
| **Ranidae** |  |  |  |  |  |  | |
| *Lithobates forreri* | 1 | - | - | - | 13 | 14 | |
| *Lithobates vaillanti* | - | 9 | - | - | - | 9 | |
| *Lithobates warszewitschii* | 20 | 3 | 19 | - | - | 42 | |
| **Number of species** | 11 | 23 | 8 | 7 | 6 | 37 | |
| **Number of individuals** | 240 | 287 | 31 | 32 | 70 | 660 | |
