# Supplementary material for: Amphibian diversity across three adjacent ecosystems in Área de Conservación Guanacaste, Costa Rica
Source: PeerJ. 2023 Nov 27;11:e16185. doi: 10.7717/peerj.16185 (PMC10688307; doi:10.7717/peerj.16185)
Supplement: Supplemental Information 4 — Compiled with data from the Museo de Zoologia at the Universidad de Costa Rica, GBIF, Arctos Collaborative Collection and this study. [file peerj-11-16185-s004.docx]

|  | |
| --- | --- |
| **Taxa** | |
| **Bufonidae** | |
| *Atelopus varius* | |
| *Bufo melanochlorus* | |
| *Crepidophryne guanacaste* | |
| *Incilius coccifer* | |
| *Incilius luetkenii* | |
| *Incilius coniferus* | |
| *Rhaebo haematiticus* | |
| *Rhinella horibilis* | |
| **Caeciliidae** | |
| *Gymnophis multiplicata* | |
| **Centrolenidae** | |
| *Espadarana prosoblepon* | |
| *Cochranella granulosa* | |
| *Hyalinobatrachium colymbiphyllum* | |
| *Hyalinobatrachium fleischmanni* | |
| *Sachatamia albomaculata* | |
| *Teratohyla pulverata* | |
| *Teratohyla spinosa* | |
| **Craugastoridae** | |
| *Craugastor andi* | |
| *Craugastor bransfordii* | |
| *Craugastor crassidigitus* | |
| *Craugastor cuaquero* | |
| *Craugastor fitzingeri* | |
| *Craugastor gollmeri* | |
| *Craugastor megacephalus* | |
| *Craugastor melanosticus* | |
| *Craugastor mimus* | |
| *Craugastor noblei* | |
| *Craugastor persimilis* | |
| *Craugastor podiciferus* | |
| *Craugastor ranoides* | |
| *Craugastor rugulosus* | |
| *Craugastor stejnegerianus* | |
| *Craugastor talamancae* | |
| *Craugastor underwoodi* | |
| **Dendrobatidae** | |
| *Oophaga pumilio* | |
| **Dermophiidae** | |
| *Gymnopis multiplicata* | |
| **Eleutherodactyldae** | |
| *Diasporus diastema* | |
| *Diasporus hylaeformis* | |
| **Hylidae** | |
| *Agalychnis callidryas* | |
| *Agalychnis saltator* | |
| *Cruziohyla sylviae* | |
| *Dendropsophus ebraccatus* | |
| *Dendropsophus micropcephalus* | |
| *Dendropsophus phlebodes* | |
| *Duellmanohyla rufioculis* | |
| *Duellmanohyla uranochroa* | |
| *Ecnomiohyla miliaria* | |
| *Hypsiboas rufitelus* | |
| *Isthmohyla psuedopuma* | |
| *Isthmohyla tica* | |
| *Scinax boulengeri* | |
| *Scinax elaeochroa* | |
| *Scinax staufferi* | |
| *Smilisca baudinii* | |
| *Smilisca puma* | |
| *Smilisca sordida* | |
| *Tlalocohyla loquax* | |
| *Trachycephalus typhonius* | |
| *Trachycephalus vermiculatus* | |
| *Tripion spinosus* | |
| **Leptodactylidae** | |
| *Engystomops pustulosus* | |
| *Leptodactylus fragilis* | |
| *Leptodactylus melanonotus* | |
| *Leptodactylus poecilochilus* | |
| *Leptodactylus savagei* | |
| **Microhylidae** | |
| *Hypopachus pictiventris* | |
| *Hypopachus variolosus* | |
| **Plethodontidae** | |
| *Bolitoglossa robusta* | |
| *Bolitoglossa striatula* | |
| *Bolitoglossa subpalmata* | |
| *Nototriton guanacaste* | |
| **Ranidae** | |
| *Lithobates forreri* | |
| *Lithobates pipiens* | |
| *Lithobates taylori* | |
| *Lithobates vaillanti* | |
| *Lithobates vivicarius* | |
| *Lithobates warszewitschii* | |
| **Rhinophrynidae** | |
| *Rhinophrynus dorsalis* | |
| **Strabomantidae** | |
| *Pristimantis cerasinus* | |
| *Pristimantis educatoris* | |
| *Pristimantis ridens* | |
