# Supplementary material for: Amphibian diversity across three adjacent ecosystems in Área de Conservación Guanacaste, Costa Rica
Source: PeerJ. 2023 Nov 27;11:e16185. doi: 10.7717/peerj.16185 (PMC10688307; doi:10.7717/peerj.16185)
Supplement: Supplemental Information 5 — Includes the total number of individuals of each species caught at each site and in total at all sites. [file peerj-11-16185-s005.docx]

|  | | | | | | | |
| --- | --- | --- | --- | --- | --- | --- | --- |
| **Taxa** | **Cacao** | **San Gerardo** | **Santa Maria** | **Maritza** | **Murcielago** | **Santa Rosa** | **Pitilla** |
| **Bufonidae** |  |  |  |  |  |  |  |
| *Incilius coccifer* | - | - | - | - | - | 2 | - |
| *Rhaebo haematiticus* | - | 4 | - | - | - | - | - |
| *Rhinella horribilis* | - | 1 | - | - | - | - | - |
| **Craugastoridae** |  |  |  |  |  |  |  |
| *Craugastor bransfordii* | 1 | 1 | - | 1 | - | - | - |
| *Craugastor crassidigitus* | 1 | - | - | - | - | - | 1 |
| *Craugastor fitzingeri* | - | 4 | - | - | - | - | - |
| *Craugastor megacephalus* | - | 3 | - | - | - | - | 2 |
| *Craugastor persimilis* | - | 3 |  | - | - | - | 3 |
| *Craugastor ranoides* | - | - | - | - | 1 | - | - |
| **Hylidae** |  |  |  |  |  |  |  |
| *Dendropsophus ebraccatus* | - | - | - | - | - | - | 3 |
| *Dendropsophus microcephalus* | - | - | - | - | - | 1 | - |
| *Duellmanohyla rufioculis* | 5 | - | - | - | - | - | - |
| *Scinax elaeochroa* | - | 2 | - | - | - | - | 2 |
| *Smilisca baudinii* | - | 1 | - | - | 1 | 2 | - |
| *Smilisca puma* | - | 1 | - | - | - | - | - |
| *Smilisca sordida* | - | 2 | - | - | - | - | 3 |
| *Tlalocohyla loquax* | - | - | - | - | - | - | 2 |
| **Leptodactylidae** |  |  |  |  |  |  |  |
| *Engystomops pustulosus* | - | - | - | - | 2 | 22 | - |
| *Leptodactylus melanonatus* | - | 1 | - | - | - | - | - |
| **Microhylidae** |  |  |  |  |  |  |  |
| *Hypopachus variolosus* | - | - | - | - | - | 2 | - |
| **Phyllomedusidae** |  |  |  |  |  |  |  |
| *Agalychnis callidryas* | - | - | - | - | - | - | 1 |
| *Agalychnis saltator* | - | - | - | - | - | - | 1 |
| **Ranidae** |  |  |  |  |  |  |  |
| *Lithobates forreri* | - | 2 | - | - | - | 1 | - |
| *Lithobates vaillanti* | - | 1 | - | - | - | - | - |
| *Lithobates warszewitschii* | 4 | - | 6 | 3 | - | - | - |
| **Number of species** | 4 | 13 | 1 | 2 | 2 | 6 | 9 |
| **Number of individuals** | 11 | 26 | 6 | 4 | 4 | 30 | 18 |
