# Supplementary material for: Amphibian diversity across three adjacent ecosystems in Área de Conservación Guanacaste, Costa Rica
Source: PeerJ. 2023 Nov 27;11:e16185. doi: 10.7717/peerj.16185 (PMC10688307; doi:10.7717/peerj.16185)
Supplement: Supplemental Information 6 [file peerj-11-16185-s006.docx]

| **TABLE S5:**  Full species list for Cacao. Compiled with data from museum records, Arctos Collaborative Collection and this study. |
| --- |
| **Taxa** |
| **Bufonidae** |
| *Atelopus varius* |
| *Incilius valliceps* |
| *Rhinella horibilis* |
| *Rhaebo haematiticus* |
| **Centrolenidae** |
| *Hyalinobatrachium colymbiphyllum* |
| *Hyalinobatrachium fleischmanni* |
| *Espadarana prosoblepon* |
| **Craugastoridae** |
| *Craugastor bransfordii* |
| *Craugastor crassidigitus* |
| *Craugastor fitzingeri* |
| *Craugastor megacephalus* |
| *Craugastor andi* |
| *Craugastor melanosticus* |
| *Craugastor ranoides* |
| *Craugastor stejnegerianus* |
| *Craugastor talamancae* |
| *Pristimantis ridens* |
| *Pristimantis cerasinus* |
| **Eleutherodactyldae** |
| *Diasporus diastema* |
| **Hylidae** |
| *Dendropsophus ebraccatus* |
| *Dendropsophus micropcephalus* |
| *Duellmanohyla uranochroa* |
| *Duellmanohyla rufioculis* |
| *Smilisca baudinii* |
| *Scinax boulengeri* |
| *Scinax elaeochroa* |
| *Tlalocohyla loquax* |
| *Isthmohyla tica* |
| **Leptodactylidae** |
| *Engystomops pustulosus* |
| *Leptodactylus melanonotus* |
| *Leptodactylus poecilochilus* |
| **Microhylidae** |
| *Hypopachus variolosus* |
| **Ranidae** |
| *Lithobates forreri* |
| *Lithobates taylori* |
| *Lithobates vaillanti* |
| *Lithobates warszewitschii* |
| **Plethodontidae** |
| *Nototriton guanacaste* |
| *Bolitoglossa robusta* |
| *Bolitoglossa subpalmata* |
